# Predicting the Efficacy of Neoadjuvant Chemotherapy for Pancreatic Cancer Using Deep Learning of Contrast-Enhanced Ultrasound Videos

**DOI:** 10.3390/diagnostics13132183

**Published:** 2023-06-27

**Authors:** Yuming Shao, Yingnan Dang, Yuejuan Cheng, Yang Gui, Xueqi Chen, Tianjiao Chen, Yan Zeng, Li Tan, Jing Zhang, Mengsu Xiao, Xiaoyi Yan, Ke Lv, Zhuhuang Zhou

**Affiliations:** 1 Department of Ultrasound, Peking Union Medical College Hospital, Chinese Academy of Medical Sciences and Peking Union Medical College, Beijing 100730, China; 2 Department of Biomedical Engineering, Faculty of Environment and Life, Beijing University of Technology, Beijing 100124, China; 3 Department of Medical Oncology, Peking Union Medical College Hospital, Chinese Academy of Medical Sciences and Peking Union Medical College, Beijing 100730, China

**Keywords:** deep learning, contrast-enhanced ultrasound, pancreatic cancer, neoadjuvant chemotherapy, prognosis prediction

## Abstract

Contrast-enhanced ultrasound (CEUS) is a promising imaging modality in predicting the efficacy of neoadjuvant chemotherapy for pancreatic cancer, a tumor with high mortality. In this study, we proposed a deep-learning-based strategy for analyzing CEUS videos to predict the prognosis of pancreatic cancer neoadjuvant chemotherapy. Pre-trained convolutional neural network (CNN) models were used for binary classification of the chemotherapy as effective or ineffective, with CEUS videos collected before chemotherapy as the model input, and with the efficacy after chemotherapy as the reference standard. We proposed two deep learning models. The first CNN model used videos of ultrasound (US) and CEUS (US+CEUS), while the second CNN model only used videos of selected regions of interest (ROIs) within CEUS (CEUS-ROI). A total of 38 patients with strict restriction of clinical factors were enrolled, with 76 original CEUS videos collected. After data augmentation, 760 and 720 videos were included for the two CNN models, respectively. Seventy-six-fold and 72-fold cross-validations were performed to validate the classification performance of the two CNN models. The areas under the curve were 0.892 and 0.908 for the two models. The accuracy, recall, precision and F1 score were 0.829, 0.759, 0.786, and 0.772 for the first model. Those were 0.864, 0.930, 0.866, and 0.897 for the second model. A total of 38.2% and 40.3% of the original videos could be clearly distinguished by the deep learning models when the naked eye made an inaccurate classification. This study is the first to demonstrate the feasibility and potential of deep learning models based on pre-chemotherapy CEUS videos in predicting the efficacy of neoadjuvant chemotherapy for pancreas cancer.

## 1. Introduction

Pancreatic cancer is a highly malignant digestive-system tumor originating from the pancreatic duct epithelium. According to the global cancer statistics in 2020, pancreatic cancer ranks seventh in the world in terms of cancer-related death causes, and the five-year survival period of patients is less than 10% [1,2]. In recent years, a promising treatment trend for borderline resectable or locally advanced pancreatic cancer is neoadjuvant therapy, which means preoperative therapy in resectable diseases as well as therapies which might lead to surgery [3]. Moreover, an early response prediction of neoadjuvant therapy would be highly desired for treatment option choices and patient management.

Imaging is a crucial tool for the evaluation of pancreatic cancer, especially for the diagnosis and treatment efficacy. Contrast-enhanced ultrasound (CEUS) could be used to assess the distribution of microvascular perfusion and display the blood vessels around the tumor. Thus, it gradually becomes an indispensable modality to facilitate pancreatic biopsy and evaluate the prognosis of chemotherapy [4,5]. Enhancement pattern is a crucial parameter for different imaging modalities. The patient prognosis of pancreas cancer is possibly revealed from different enhancement patterns in recent years [6,7]. The enhancement pattern, such as hypo- or hyper-enhancement, of focal pancreatic lesions is compared with the adjacent relatively normal pancreas tissue. Hypo-enhancement means the extent of enhancement of the lesion is lower than normal tissues and hyper-enhancement vice versa. For CEUS, previous studies also demonstrated that patients with hypo-enhancement pattern had shorter survival time compared with those with iso- or hyper-enhancement pattern [8,9]. Those studies also indicated that CEUS had a great potential to predict the efficacy of pancreatic cancer therapy.

With the development of deep learning technology, it has been widely used in clinical medicine [10]. By using a large amount of medical data and artificial intelligence algorithms, deep learning technology can automatically identify lesions in medical images and predict the development trend of diseases to provide treatment recommendations for doctors [11], improving the accuracy and speed of medical diagnosis and reducing the workload of doctors. In medical imaging and clinical chemotherapy, deep learning helps doctors more accurately assess the side effects and risks of chemotherapy [12,13], as well as determine the best treatment time and dose, by analyzing large amounts of case data to predict patients’ conditions and responses to chemotherapy drugs, thus guiding doctors to establish personalized treatment strategies. It provides patients with a more accurate and personalized treatment plan.

Deep learning could efficiently extract temporal and spatial features of images, which may even not be perceived by naked eyes or human-defined features [14,15]. For pancreatic diseases, deep learning algorithms based on CEUS were developed to predict preoperative aggressiveness of pancreatic neuroendocrine neoplasms and differential diagnosis of solid tumors [16,17]. However, these attempts only used specific CEUS frames, rather than videos, and a large quantity of potential spatiotemporal features within CEUS videos may be missed in consequence. Clinically, both borderline resectable and locally advanced pancreatic cancer patients require chemotherapy, but since some patients are not sensitive to chemotherapy, we propose to predict the efficacy of chemotherapy for pancreatic cancer in order to avoid patient suffering or delayed treatment to construct personalized treatment plans. However, the feasibility of using CEUS and deep learning to predict the effectiveness of chemotherapy for pancreatic cancer remains unknown. We hypothesized that deep learning models based on CEUS videos may be used to directly predict the effectiveness of pancreatic cancer chemotherapy. The objective of this study was to evaluate the feasibility of deep learning models based on CEUS videos for predicting the efficacy of chemotherapy for pancreatic cancer.

In this study, we proposed a deep learning strategy based on CEUS videos to predict the efficacy of neoadjuvant chemotherapy for pancreatic cancer. Clinical CEUS videos of patients with pancreatic cancer were collected before chemotherapy to validate the feasibility of the proposed deep learning models.

## 2. Materials and Methods

### 2.1. Patient Enrollment

This retrospective study was approved by the Institutional Review Board of Peking Union Medical College Hospital, Beijing, China. Patients with pathologically confirmed pancreatic ductal adenocarcinoma were enrolled for the study. Resectability was classified as borderline resectable or locally advanced pancreatic cancer after a multidisciplinary consultation at Peking Union Medical College Hospital, Beijing, China, a high-volume center for pancreatic diseases. All patients received at least four courses (no more than eight) of neoadjuvant chemotherapy of nab-paclitaxel and tegafur, gimeracil and oteracil potassium (S-1) before surgery.

**Figure 1:**
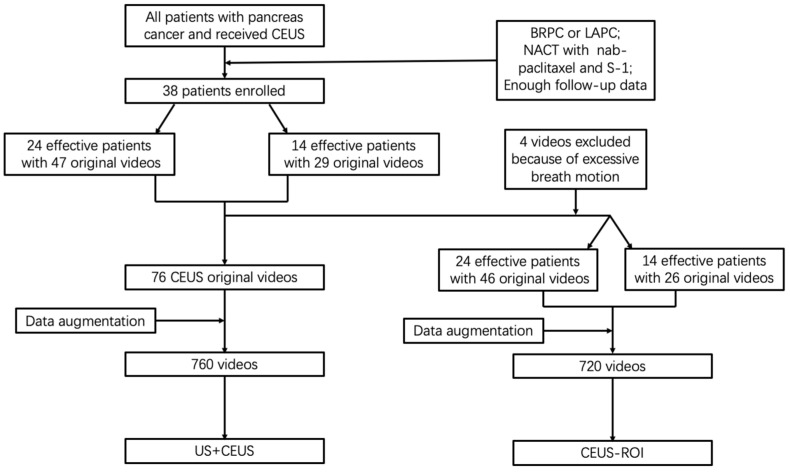
Flow chart of patient enrollment and data preprocessing. BRPC: borderline resectable pancreatic cancer; LAPC: locally advanced pancreatic cancer; NACT: neoadjuvant chemotherapy; S-1: tegafur, gimeracil and oteracil potassium; US: ultrasound; CEUS: contrast-enhanced ultrasound; ROI: region of interest.

The patients were followed-up regularly at our medical center. Abdominal contrast-enhanced computerized tomography scans were performed before the treatment and after every two courses of chemotherapy to evaluate the tumor size. The resectability was assessed by National Comprehensive Cancer Network (NCCN v2.2018) resectability criteria for pancreatic cancer, after a multidisciplinary consultation. For patients with locally advanced pancreatic cancer, neoadjuvant chemotherapy was considered clinically effective if the disease was partial remission (PR), or stable disease (SD) after 6 months, judged by the Response Evaluation Criteria In Solid Tumors (RECIST) 1.1. For patients with borderline resectable pancreatic cancer, the neoadjuvant chemotherapy was considered clinically effective if the operation after the chemotherapy achieved R0 resection. There were 38 patients (24 effective and 14 ineffective) included in this study, and 76 CEUS videos (47 effective and 29 ineffective) were obtained (Figure 1).

### 2.2. Ultrasound and CEUS

The ultrasound (US) and CEUS examinations were performed 1–7 days before the first course of chemotherapy for each patient. The examination was conducted by two well-trained radiologists with over 10 years’ experience in US and over 5 years’ experience in CEUS. The parameter adjustment of the ultrasound scanner and the contrast operation followed the manufacturer’s instructions and clinical routine. The patients were instructed to stay as still as possible to continuously observe the dynamic perfusion process of the lesion in real time. The enhanced dynamic videos were stored in the AVI format.

**Figure 2:**
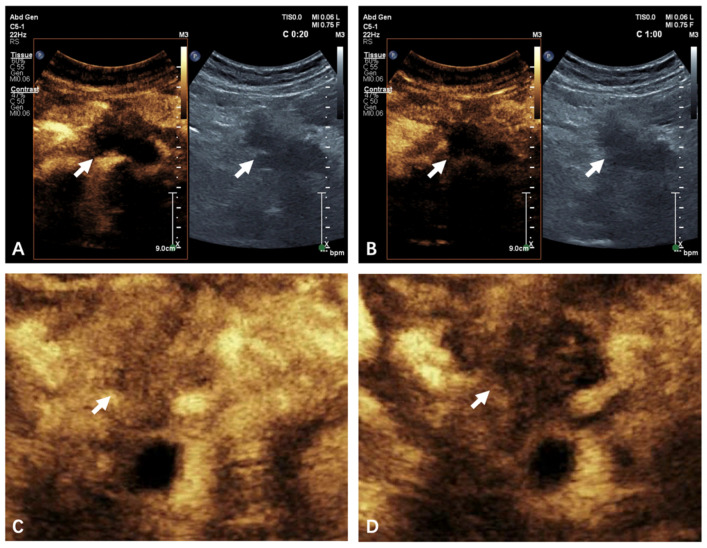
US+CEUS and CEUS-ROI images. (**A**,**B**) An example of typical US+CEUS frames of 20 s (arterial phase) (**A**) and 60 s (venous phase) (**B**) of a patient with ineffective treatment outcome, showing hypo-enhancement pattern. (**C**,**D**) An example of typical CEUS-ROI frame of 20 s (arterial phase) (**C**) and 60 s (venous phase) (**D**) of a patient with effective treatment outcome, showing iso-enhancement pattern. The white arrow indicates pancreas cancer. US: ultrasound; CEUS: contrast-enhanced ultrasound; ROI: region of interest.

A lesion may have been examined for multiple times, both transversely and longitudinally in the maximum possible layer. The CEUS process was divided into the arterial phase and the venous phase, defined as <30 s and 31–120 s after the injection of the contrast agent, respectively. Typical CEUS cines in both phases were illustrated in Figure 2A,B. Enhancement pattern was classified as iso-enhancement or hypo-enhancement compared with the normal pancreatic parenchyma by naked eyes [5]. Videos with an iso-enhancement pattern were classified as effective by the naked eye, denoted as NE-EF, and NE-INEF vice versa.

### 2.3. CEUS Video Preprocessing

Each frame of the CEUS videos contained one US image and one CEUS image correspondingly (Figure 2A,B). The average frame rate was 14.36 frames per second (FPS), and the average frame number was 1507 for each video.

**Figure 3:**
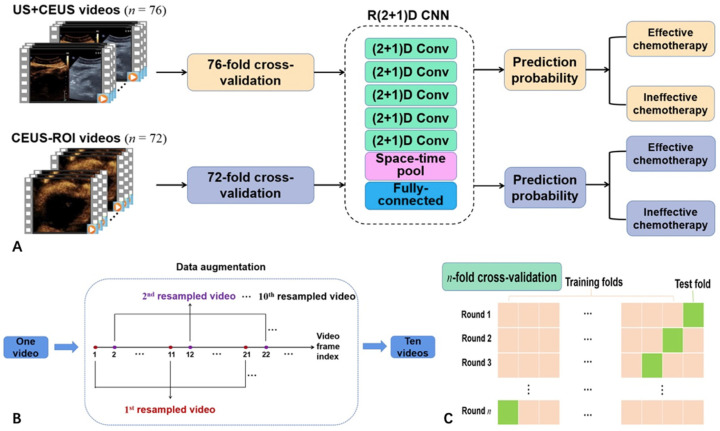
(**A**) Flow chart of the proposed deep learning method. (**B**) Data augmentation. (**C**) *n*-fold cross-validation.

Considering that the amount of available videos for training was small, the time span of the videos was long, and the numbers of the positive and negative samples were unbalanced, it was necessary to augment the experimental data. The method of frame sampling was used for video reorganization and data augmentation. According to the sequence of frames, samples were taken every 10 frames [18]. Therefore, one video could be divided into 10 groups of different video segments. For instance, the first video segment contained the 1st, 11th, 21st, … frames of the original video, and the second video contained the 2nd, 12th, 22nd, … frames of the original video. In addition, considering the complete cycle of blood supply, all the video segments covered the entire phase of the CEUS scan, including the arterial and venous phase. After data augmentation, we obtained 760 videos, denoted as US+CEUS. These videos were used for the first deep learning model (Figure 3).

We then built another deep learning model by defining the region of interest (ROI). Among the 76 videos before data augmentation, 4 were excluded due to relatively excessive breath motion. Finally, 72 videos (46 effective and 26 ineffective) were selected for analyzing only CEUS images. Considering the similarity of the images in late venous phase, we only used the first 60 s of the original videos. The average frame rate was 14.02 FPS and the average frame number was 855 for each video in this group. For the 72 videos, we selected a rectangular ROI within the CEUS image to obtain 72 ROI videos (Figure 2C,D). The ROI was selected to cover the entire lesion and part of the normal pancreas, inevitably including some nearby tissues. After similar process of data augmentation, we obtained another 720 videos, denoted CEUS-ROI (Figure 3).

### 2.4. Network Structure

Figure 3A shows the flow chart of the proposed deep learning method. Preprocessed pancreatic CEUS videos were used as the input to a pre-trained convolutional neural network (CNN). The network automatically fine-tuned model weights and biases, and finally yielded a prediction probability. We aimed to automatically extract multi-level information related to dynamic microbubble perfusion in pancreatic cancer from CEUS videos with deep learning, so three-dimensional (3D) CNN was required. However, conventional 3D CNNs had large network parameters and low computational efficiency. In order to reduce network parameters and improve computational efficiency, we used a pre-trained R(2 + 1)D CNN [19]. The initial R(2 + 1)D CNN was trained on the kinetics dataset [19]. The kinetics dataset contained 650,000 video clips covering 400 human action classes.

The R(2 + 1)D CNN could improve the efficiency of the 3D CNN model by replacing the 3D convolution filter with a combination of two-dimensional (2D) spatial convolution and one-dimensional (1D) temporal convolution. The R(2 + 1)D module decomposed the 3D convolution into two independent, continuous convolutions: one was the 2D spatial convolution, and the other was the 1D temporal convolution. The model was first convolved in the spatial dimension, and then convolved separately in the temporal dimension. We could add nonlinear rectification such as rectified linear unit (ReLU) between the 2D and 1D convolutions. Compared to 3D CNN, this would double the number of nonlinearity, but the number of parameters to be optimized was the same, reducing the computational complexity of 3D convolution. In addition, the decomposition into two convolutions made the optimization process easier and produced less training loss and testing loss in practice.

The global spatiotemporal average pooling was applied to the final convolution, and then the final classification was performed by a fully connected layer. The encoding part adopted a lightweight residual network structure, and the network input was sized [8 (batch size) × 3 (channels) × 64 (frames) × 112 (frame width) × 112 (frame height)]. For the training set, the batch size was 8. For the test set, the batch size was 10, and the output layer was changed to 1.

### 2.5. Experimental Configuration

Our experiments were conducted on a graphics workstation with Intel(R) Xeon Gold 6132 CPU@2.60 GHz 2.59 GHz, and NVIDIA TITAN RTX 24 G. Python 3.8 and Pytorch 1.12 were chosen as the deep learning framework. In this study, the mean squared error (MSE) loss function, stochastic gradient descent (SGD) optimizer, and an initial learning rate of 10–4 were used. The CNN models were analyzed using 76-fold cross-validation for US+CEUS and 72-fold cross-validation for CEUS-ROI. For the 760 US+CEUS videos, 10 augmented videos with the same data label (corresponding to one original video) were selected as the test sets, and the remaining 750 videos were used as the training sets; this process was repeated for 76 times. The 720 CEUS-ROI videos were analyzed similarly, and the process was repeated for 72 times (Figure 3C). For each time of cross-validation, 20 epochs of training were conducted, and the model with the lowest training loss was saved; then, the 10 test sets were input into the saved model to obtain 10 prediction probabilities.

### 2.6. Statistical Analysis

To evaluate the classification performance of the two CNN models, the area under the curve (AUC) was computed by analyzing the prediction probabilities of the 760 US+CEUS videos or the 720 CEUS-ROI videos. Best performance value was also identified, at which the sum of true positive rate and true negative rate (Youden’s index) achieved highest. Metrics of accuracy, recall, precision, and F1 score were also computed.

The average of each prediction probability within a test set (10 videos) was then calculated. And the final prediction for each original video (76 or 72 in two deep learning models) was output as effective (DL-EF), if the average was larger than the best performance value, which is the best performance value when the Youden’s index is the highest; otherwise it was ineffective (DL-INEF). Kappa tests were conducted and the Matthews correlation coefficient (MCC), classification success index (CSI), and geometric mean (G-mean) were calculated for consistency analyses between the two deep learning models for the 72 original videos according to the final prediction results.

Descriptive factors were summarized as mean with standard deviation. Pearson’s Chi-square tests and Student’s *t*-tests were conducted for categorical and continuous factors, respectively. A two-sided *p* value < 0.05 was considered statistically significant. Statistical Product Service Solutions (SPSS) 26.0 (IBM, Armonk, NY, USA) was used for statistical analysis.

## 3. Results

### 3.1. Clinical and Imaging Features of Patients

The clinical and imaging features of all patients were listed in Table 1. There was no significant difference in terms of onset age and gender between the two groups. The lesions were scattered in all parts of the pancreas. Color Doppler blood flow signals could be detected among only eight (21%) patients, including six in the effective group and two in the ineffective group. The enhancement patterns also showed no significant difference between the two groups, even though the ratio of iso-enhancement was relatively higher in the effective group (54% vs. 36%).

### 3.2. Performance of the Two Deep Learning Strategies

**Figure 4:**
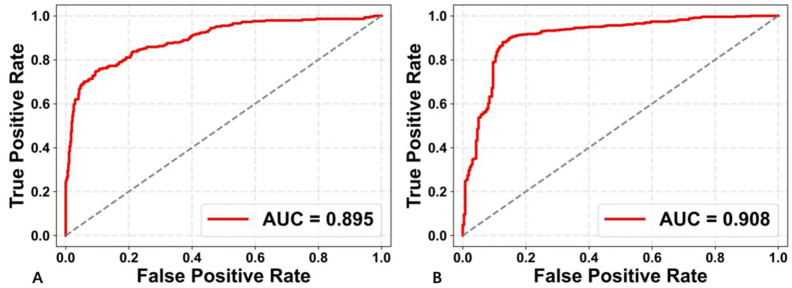
ROC curves obtained by using the two deep learning strategies with US+CEUS (**A**) and CEUS-ROI (**B**).

The performance metrics of the proposed method was illustrated in Table 2. The receiver operating characteristic (ROC) curves were shown in Figure 4. The AUCs obtained by the proposed method with US+CEUS and CEUS-ROI were 0.895 and 0.908, respectively. Compared to US+CEUS, CEUS-ROI yielded a slightly better performance in all the metrics.

### 3.3. Performance of Different 3D CNN Models

Table 3 shows the performance of different 3D CNN models for pancreatic cancer chemotherapy efficacy prediction on the CEUS-ROI datasets. The results show that the prediction effect using the R(2 + 1)D CNN model is better than that using the R3D CNN model [19].

### 3.4. Prediction of Each Original Video by US+CEUS

**Figure 5:**
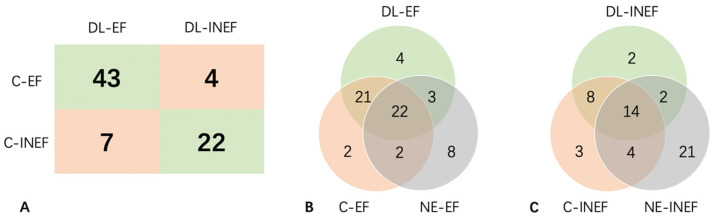
Prediction of each original video by US+CEUS. (**A**) 2 × 2 contingency table of results of final predictions of DL and in clinical practice. (**B**) Venn Diagram of the number of cases that was classified as C-EF, NE-EF, and DL-EF. (**C**) Venn Diagram of the number of cases that was classified as C-INEF, NE-INEF, and DL-INEF. C-(IN)EF: clinically (in)effective; DL-(IN)EF: deep learning predicted (in)effectiveness; NE-(IN)EF: naked eye predicted (in)effectiveness.

The best performance value was 0.5313, when Youden’s index achieved highest. After calculating the average of each video within a data set, a total of 11 videos were diagnosed incorrectly, thus the sensitivity and specificity of this deep learning strategy achieved 91.5% and 75.9%, respectively (Figure 5A).

In the clinically effective (C-EF) group, 24 (51.1%) videos were in iso-enhancement pattern and were designated as NE-EF. Among them, 22 (91.7%) were also correctly diagnosed in the deep learning model. Moreover, the deep learning model additionally identified 21 (44.7%) videos, even though their CEUS patterns were hypo-enhancement. Notably, there were still three erroneous cases when both the deep learning model and naked eye misdiagnosed them as effective (Figure 5B,C).

In the clinically ineffective (C-INEF) group, 14 (48.3%) were diagnosed correctly by both the deep learning model and naked eye. The deep learning model could distinguish another 8 (27.6%) in the C-INEF group and 29 (38.2%) in total, when the naked eye made mistakes (Figure 5B,C).

### 3.5. Prediction of Each Original Video by CEUS-ROI

**Figure 6:**
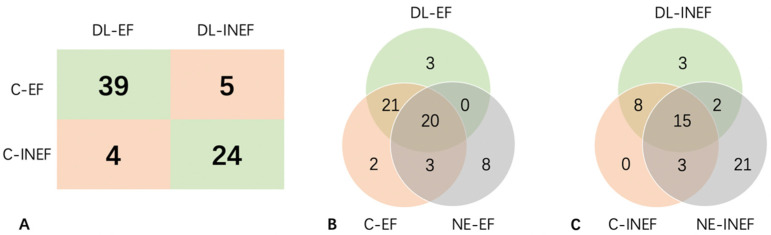
Prediction of each original video by CEUS-ROI. (**A**) 2 × 2 contingency table of results of final predictions of DL and in clinical practice. (**B**) Venn diagram of the number of cases that was classified as C-EF, NE-EF, and DL-EF. (**C**) Venn diagram of the number of cases that was classified as C-INEF, NE-INEF, and DL-INEF. C-(IN)EF: clinically (in)effective; DL-(IN)EF: deep learning predicted (in)effectiveness; NE-(IN)EF: naked eye predicted (in)effectiveness.

Youden’s index was the highest when the predicted probability was 0.6062. The sensitivity and specificity achieved 88.6% and 85.7%, respectively (Figure 6A). A total of 29 (40.3%) videos (21 effective and 8 ineffective) could be clearly distinguished by the deep learning model when naked-eye judgement was wrong. Another two videos from the effective group were misclassified by either the deep learning model or naked eye (Figure 6B,C).

The Kappa value, the MCC value, the CSI value, and the G-mean value of the results from the two deep learning models were 0.640, 0.645, 0.769, and 0.694, respectively. Four videos were wrongly classified by both of the deep learning models. One of the lesions involved the celiac trunk and hepatic artery. Since the above arteries were partly visible in the image, the recognition of the lesion boundary may be affected. In another video, a large area of acoustic attenuation was closely attached to the lesion, which may also affect the judgment of the lesion’s boundary. In the third video, the total brightness of the image fluctuated with breathing to some extent. And no evident limitations were observed from the fourth video, in terms of image quality and the lesion itself.

## 4. Discussion

In this study, we proposed a deep learning strategy based on CEUS videos to predict the efficacy of neoadjuvant chemotherapy for pancreas cancer. Two deep learning models achieved high performance with AUCs of 0.892 and 0.908. These models could correctly classify over 40% videos in addition to naked eye’s prediction, and had great potentials in clinical use. To the best of our knowledge, this study is the first to investigate the feasibility of deep learning models based on CEUS in predicting the efficacy of neoadjuvant chemotherapy for pancreas cancer.

Compared with contrast-enhanced computed tomography (CT) and contrast-enhanced magnetic resonance imaging (MRI), which could only produce four kinds of typical images in four enhancement phases, corresponding to the non-enhancement phase, the arterial phase, the portal phase, and the delayed phase, the CEUS scan would produce a large number of continuous images for one lesion. Therefore, CEUS may better reflect the continuous change in the enhancement condition of the lesion [5,20,21]. However, CEUS is operator-dependent. Using deep learning models based on CEUS may provide an end-to-end scheme to characterize the response of pancreatic cancer to chemotherapy.

CEUS could display perfusion characteristics of solid tumors and is gradually becoming an important imaging approach clinically. To better interpret CEUS, deep learning algorithms have been gradually applied in recent years, mostly based on specific CEUS frames or time-intensity curves (TICs) [22]. Under these circumstances, mass data about spatiotemporal information in CEUS videos may still be omitted. Only few attempts successfully built deep learning models based on CEUS videos, mainly for liver cancers, breast cancers and carotid plaques [23,24,25,26]. To the best of our knowledge, this paper is the first report of deep learning models based on CEUS videos for the prognostic prediction of pancreatic cancer. Directly using CEUS videos of pancreas lesions was challenging, due to inevitable breath motion, gastrointestinal gas, and complicated anatomical structures nearby. In this study, we built two deep learning models. The first model used CEUS+US videos, which contained abundant information of other organs. Moreover, the other one only used defined ROIs of the CEUS part, which paid attention mainly to the blood perfusion of the lesion itself. The results of Kappa consistency test indicated acceptable consistency, and the CEUS-ROI yielded slightly better performance. However, it is worth mentioning that several cases were misclassified by both models, possibly because of the incorrect identification of the lesion’s margin.

Enhancement pattern was regarded as the criteria of naked-eye evaluation; however, the accuracy was less than 60% in this study. The deep learning model could correctly classify another ~40% videos when naked eye made mistakes. This reflected the advantages of deep learning models in CEUS interpretation. The deep learning model could identify some hidden features, which may not be perceived by human eyes [27]. Since the preprocessing of the original CEUS videos is not hard, it is promising for the wide application of deep learning models in clinical settings.

The prognostic evaluation of pancreatic cancer was also a complicated clinical issue. For patients with similar pretreatment images, therapeutic effects may be highly influenced by disease stages and different treatment modalities, including surgery, radiotherapy, neoadjuvant chemotherapy, and targeted therapy. In this study, all patients were with borderline resectable or locally advanced pancreatic cancer, and received the same chemotherapy regimen. All patients received regular follow-up and had enough follow-up imaging results to be evaluated by RECIST. Other clinical and imaging features in Table 2 also showed no significant differences between the two groups. Therefore, those relatively strict restrictions could reduce the impact of other confounding factors and better reflect the value of CEUS videos, but the included cases were limited on the other hand. In future studies, patients received different therapies can also be considered to validate the universality of the deep learning models.

The study has some limitations. First, since we made tight restrictions on several clinical factors, and even though we conducted a data augmentation procedure, the sample size was still relatively small. Additionally, there was an imbalance in different prognosis. Additional data are needed to verify the performance of the models in future work. Second, the interpretability of the deep learning model was limited and this may be a common pitfall in the field of deep learning-based image classification. Third, our model only used CEUS videos and lacked clinical parameters and other imaging modalities. Routine clinical factors, such as tumor biomarkers would also be added in future studies.

## 5. Conclusions

Deep learning is a promising solution to quantitative assessment of CEUS videos for pancreatic solid diseases. Deep learning models reached high performance and had great potential to provide supplementary information about prognosis prediction on pancreas cancer neoadjuvant chemotherapy. Our study is the first to use deep learning models based on pancreatic CEUS videos to predict chemotherapy efficacy for pancreatic cancer. The AUC value of the deep learning model was 0.908, indicating a satisfying performance in predicting the chemotherapy efficacy of patients. Due to the limited number of datasets, data augmentation and cross-validations were used in this work. The findings of this study show the potential of deep learning models based on CEUS videos in predicting the efficacy of neoadjuvant chemotherapy for pancreatic cancer.

## Figures and Tables

**Table 1:** Clinical and imaging features.

	Effective (*n* = 24)	Ineffective (*n* = 14)	*p*
Age (years)	57.8 ± 7.2	55.9 ± 10.7	0.58
Gender			0.56
Male	16	8	
Female	8	6	
Tumor size, cm	4.47	4.11	0.40
Location			0.62
Head and neck ^1^	10	7	
Body and tail	14	7	
CA199 (U/mL)	672.4 ± 1241.3	460.1 ± 726.0	0.27
Doppler blood flow signals			0.68
Positive	6	2	
Negative	18	12	
Enhancement pattern			0.27
Iso-enhanced	13	5	
Hypo-enhanced	11	9	

^1^ Including uncinate process.

**Table 2:** The performance metrics of the two proposed deep learning methods.

	AUC	Accuracy	Recall	Precision	F1 Score
US+CEUS	0.895	0.829	0.759	0.786	0.772
CEUS-ROI	0.908	0.864	0.930	0.866	0.897

**Table 3:** Comparison of the performance in predicting chemotherapy efficacy in pancreatic cancer using different 3D CNN models with the CEUS-ROI datasets.

	AUC	Accuracy	Recall	Precision	F1 Score
R(2 + 1)D	0.908	0.864	0.930	0.866	0.897
R3D [19]	0.889	0.814	0.612	0.828	0.704

## Data Availability

The raw data supporting the conclusions of this article may be provided upon reasonable requests for scientific research purposes.
